# 3D Printing Technologies for Flexible Tactile Sensors toward Wearable Electronics and Electronic Skin

**DOI:** 10.3390/polym10060629

**Published:** 2018-06-07

**Authors:** Changyong Liu, Ninggui Huang, Feng Xu, Junda Tong, Zhangwei Chen, Xuchun Gui, Yuelong Fu, Changshi Lao

**Affiliations:** 1 Additive Manufacturing Institute, College of Mechatronics & Control Engineering, Shenzhen University, Shenzhen 518060, China; liuchangyong@aliyun.com (C.L.); 15875539543@163.com (N.H.); 15258511107@163.com (F.X.); tong_oooo@163.com (J.T.); chen@szu.edu.cn (Z.C.); fuyl@sina.cn (Y.F.); 2 Guangdong Provincial Key Laboratory of Micro/Nano Optomechatronics Engineering, Shenzhen University, Shenzhen 518060, China; 3 State Key Laboratory of Optoelectronic Materials and Technologies, School of Electronics and Information Technology, Sun Yat-Sen University, Guangzhou 510275, China; guixch@mail.sysu.edu.cn

**Keywords:** 3D printing, tactile sensors, wearable electronics, electronic skin

## Abstract

3D printing has attracted a lot of attention in recent years. Over the past three decades, various 3D printing technologies have been developed including photopolymerization-based, materials extrusion-based, sheet lamination-based, binder jetting-based, power bed fusion-based and direct energy deposition-based processes. 3D printing offers unparalleled flexibility and simplicity in the fabrication of highly complex 3D objects. Tactile sensors that emulate human tactile perceptions are used to translate mechanical signals such as force, pressure, strain, shear, torsion, bend, vibration, etc. into electrical signals and play a crucial role toward the realization of wearable electronics and electronic skin. To date, many types of 3D printing technologies have been applied in the manufacturing of various types of tactile sensors including piezoresistive, capacitive and piezoelectric sensors. This review attempts to summarize the current state-of-the-art 3D printing technologies and their applications in tactile sensors for wearable electronics and electronic skin. The applications are categorized into five aspects: 3D-printed molds for microstructuring substrate, electrodes and sensing element; 3D-printed flexible sensor substrate and sensor body for tactile sensors; 3D-printed sensing element; 3D-printed flexible and stretchable electrodes for tactile sensors; and fully 3D-printed tactile sensors. Latest advances in the fabrication of tactile sensors by 3D printing are reviewed and the advantages and limitations of various 3D printing technologies and printable materials are discussed. Finally, future development of 3D-printed tactile sensors is discussed.

## 1. Introduction

Epidermal electronic system or electronic skin has been introduced as a multifunctional electronic system to detect the physiological signals including temperature, pressure, strain and electrophysiological signals by integrating electrodes, various sensors, power supply and communication components onto a flexible and stretchable substrate [1]. These wearable artificial skin systems have important applications in biomedical engineering and health monitoring [1,2,3,4,5,6,7,8,9,10,11,12,13]. Among all the components incorporated in an epidermal electronic system, flexible tactile sensors able to detect force, pressure, strain, shear, torsion, bend and vibration produced by human subtle touch, contact and movement play a very important role [10,14,15]. They require high sensitivity with the minimum detectable pressure as low as ~5 Pa [16,17]. In addition to sensitivity, flexibility, repeatability/reproducibility and frequency responses are also important for the long-term and real-time monitoring of movement [18]. To date, many researchers have introduced various novel sensing materials with specially designed micro/nanostructures and fabrication schemes to fulfill the requirements [16,18,19,20,21,22,23,24,25,26,27,28,29,30,31,32,33,34,35,36,37,38,39,40,41,42,43,44]. These efforts have greatly advanced the field of flexible and wearable tactile sensors.

In the past few years, 3D printing has attracted a lot of attention due to its manufacturing simplicity, attainable complexity of printed parts, increasing types of printable materials, capability of fabricating multi-material objects, tailored microstructures and declining cost. Due to these advantages, 3D printing has been applied in the fabrication of various functional devices including energy storage devices (lithium-ion batteries [45,46,47,48,49,50,51,52,53,54], super-capacitors [55] and fuel cells [56,57]), electronic devices [58,59,60], biomedical devices [61,62,63,64], and various sensors [65]. As for flexible tactile sensors, 3D printing has proven to be an easy, cost-effective and scalable fabrication technique as an alternative to conventional fabrication processes, most of which are complex, high-cost and time-consuming. This review attempts to summarize 3D printing applications in tactile sensors. Latest advances on the printing processes and printable materials are reviewed. Advantages and limitations of various 3D printing processes and future development of 3D-printed tactile sensors are discussed.

## 2. A Brief Overview of Tactile Sensors

### 2.1. Functional Requirements of Tactile Sensors

Tactile sensors are used to emulate human tactile perception such as pressure, shear, bend, torsion, strain and vibration signals with pressure range of 5 Pa–100 KPa and vibration frequency up to ~400 Hz [66]. When tactile sensors are subjected to external loads, mechanical signals are translated into electrical signals that can be detected by an external analyzer. To replicate the functionality of human skin, the requirements of wearable tactile sensors include: (1) flexibility, bendability and stretchability so that the tactile sensors are skin-attachable and wearable; (2) high sensitivity so that minimal pressure, strain, shear and torsion can be detected; (3) wide pressure range; (4) high repeatability and reproducibility over cyclic loading and unloading; (5) good linear input–output characteristics; and (6) fast response to dynamic loads. These requirements have posed great challenges for materials and fabrication technologies. Facile, low cost, reproducible and scalable fabrication technologies have always been a major challenge.

### 2.2. Sensing Mechanism of Flexible Tactile Sensors

**Figure 1:**
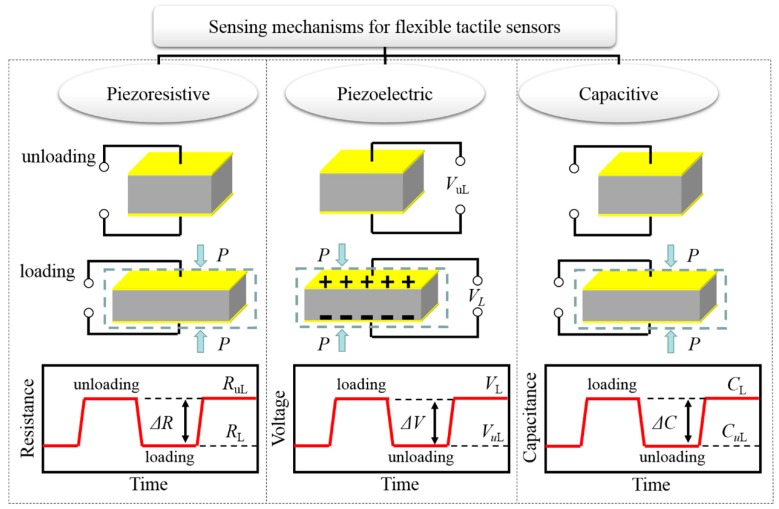
Sensing mechanisms for tactile sensors.

Pressure sensing can be achieved through four main sensing mechanisms: piezoresistive, piezoelectric, capacitive and field-effect transistor. Among the four sensing mechanisms, conventional silicon metal-oxide semiconductor field-effect transistor based sensors have a high sensitivity, however they are unsuitable for flexible devices due to its inherent rigidity [22]. To address this issue, organic field-effect transistor (OFET) based on stretchable polymers was proposed [67,68,69,70,71]. Very recently, an intrinsically stretchable polymer OFET array with density of 347 transistors per square centimeter was demonstrated [72]. The transistors exhibited charge-carrier mobility comparable to that of amorphous silicon and varied slightly when subjected to 100% strain for 1000 cycles. However, to date, it is still very difficult to fabricated OFET-based tactile sensors via 3D printing. Thus, 3D-printed tactile sensors have mainly adopted the three other sensing mechanisms. Therefore, we only focus on these three types of tactile sensor and OFET-based sensors are not described in this review. The basic principles of piezoresistive, capacitive and piezoelectric sensing mechanisms are shown in Figure 1.

#### 2.2.1. Piezoresistive Sensing

As for Piezoresistive sensing, a sensing layer made from piezoresistive materials translates mechanical signals into resistive/conductance change and measured by an external circuit. Conventionally, piezoresistive metal or semiconductor foil patterns are used as the sensing element for commercial strain gauges. Despite the high sensitivity and low cost, these commercial strain gauges are fixed directional sensors and only able to measure very small strains (usually less than 5%) [18]. Thus, they are not suitable for wearable tactile sensors that are multifunctional, multidirectional and require high sensitivity in a wide pressure range. To meet the requirements of flexible tactile sensors for wearable electronics, novel sensing element based various nanomaterials are required. To date, many types of nanomaterials including nanowires (gold and silver) [23,26,73], carbon nanotubes (CNTs) [17,18,19,44,74,75,76,77,78,79,80], polymer micro/nanostructures [16,81], metal nanoparticles [74,82,83,84] and graphene [20,21,22,25,27,28,30,31,32,33,34,36,38,39,41,42,43,44,66,75,82,85,86,87,88,89,90,91,92,93,94,95] have been applied to the design of flexible tactile sensors. Remarkable progress has been made in improving the sensitivity, flexibility, stretchability, frequency response and durability of tactile sensors. For example, Amjadi et al. [23] fabricated a strain sensor by embedding silver nanowire (AgNWs) networks between two polydimethylsiloxane (PDMS) layers. The strain sensor showed excellent piezoresistivity with tunable gauge factor (2–14) and high stretchability up to 70%.

#### 2.2.2. Piezoelectric Sensing

Piezoelectric effect is a widely used mechanism to fabricate various sensors by converting the change of pressure, acceleration, force, strain, etc. into measurable electrical quantity. Conventionally, piezoelectric ceramics, single crystals and ceramic/polymer composites [96,97,98,99,100,101] were used as the sensing materials. However, inorganic ceramics are inherently brittle and not suitable for flexible tactile sensors. Hence, polymer-based piezoelectric materials such as poly(vinylidene fluoride) (PVDF) and copolymerized poly(vinylidene fluoride-trifluoroethylene) (PVDF-TrFE) have been used for flexible tactile sensors [102,103]. For example, arrays of highly aligned piezoelectric nanofibers of PVDF and PVDF-TrFE fabricated via electrospinning were applied to fabricate flexible pressure sensors and a pressure sensitivity of over 1.1 V·KPa^−1^ was achieved [102]. In addition, many novel hybrid nanocomposites were developed such as PbTiO_3_/graphene heterostructure [104] and composite films of alkaline niobate K_0.485_Na_0.485_Li_0.03_NbO_3_ (KNLN) powders aligned in PDMS matrix [105]. However, available piezoelectric polymers are still very limited and functional materials with both excellent piezoelectric properties and sufficient stretchability require further development.

#### 2.2.3. Capacitive Sensing

Capacitive sensing relies on the capacitance change of the dielectric layer sandwiched by conductive electrodes. Pressure or strain-induced deformation that brings the electrodes closer results in the increase of capacitance, and vice versa [106,107,108]. As an example, Lipomi et al. [107] developed a capacitive pressure and strain sensor composed of a silicone elastomer Ecoflex layer as dielectric and two conductive single-walled carbon nanotubes-coated (SWCNTs) PDMS electrodes. Using these soft elastomers as dielectric and conductive electrodes, capacitive tactile sensors with required stretchability can be obtained.

For a capacitive tactile sensor, the structure of elastomeric dielectric layer is crucial to its performance. Originally, the dielectric layer was made of solid elastomers. However, these sensors suffered from low pressure sensitivity and serious hysteresis due to the viscoelastic properties of elastomers. To address this issue, elastomeric dielectric layers with surface microstructures or 3D porous structures were proposed [109]. The existence of voids in these structures could enable the dielectric layer to elastically and reversibly deform on the application of external pressure or force [6,70,110,111,112,113,114,115,116,117,118]. It was found that the sensitivity can be greatly improved, and hysteresis can be greatly reduced. For example, Mannsfeld et al. [106] fabricated microstructured PDMS films using a replica molding process. Capacitive sensors were fabricated using the microstructured PDMS film as the dielectric layer. Greatly improved sensitivity was achieved (0.55 KPa^−1^ vs. 0.02 KPa^−1^ for unstructured dielectric layer). In addition, stretchable electrodes are also required for capacitive tactile sensors. Percolation electrodes based on various carbon nanomaterials and metal nanoparticles offer both excellent electrical conductivity and stretchability. By combining various microstructured and porous dielectric elastomeric layer and stretchable percolation electrodes, the performance of capacitive tactile sensors have been greatly enhanced. For example, Kwon et al. [110] fabricated a capacitive pressure sensor composed of 3D microporous Ecoflex dielectric layer and two CNTs-Ecoflex nanocomposite films as percolation electrodes. A pressure sensitivity of 0.601 KPa^−1^ was obtained.

## 3. 3D printing Applications in Tactile Sensors

### 3.1. A Brief Overview of 3D Printing

**Figure 2:**
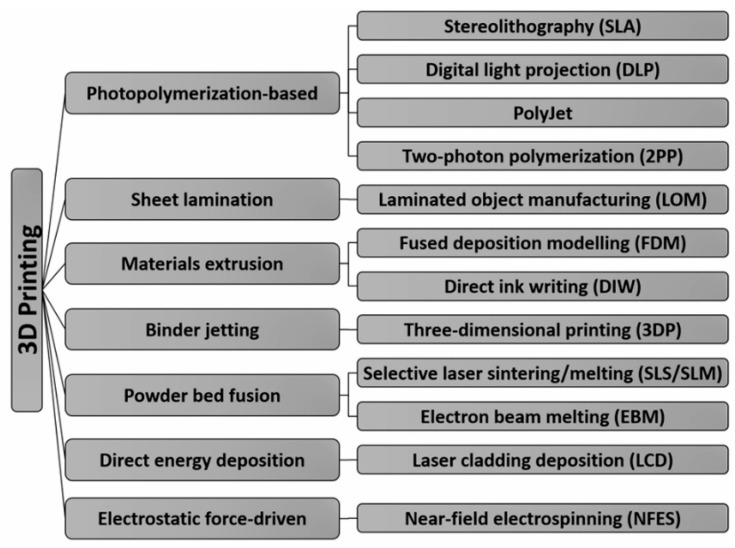
3D printing technologies.

3D printing refers to a group of technologies that fabricate three-dimensional objects directly from computer-aided design (CAD) models in a layer-by-layer fashion [119,120,121]. With 3D printing, nearly any arbitrarily complex object can be produced in a simple and low-cost manner. To date, many types of 3D printing technologies have been developed. These technologies are usually categorized into photopolymerization-based, sheet lamination-based, materials extrusion-based, power bed fusion-based, direct energy deposition and binder jetting-based technologies. Commonly used 3D printing technologies are shown in Figure 2. There have been many comprehensive reviews on 3D printing and its applications in various fields including metallic parts [120], biomedical engineering [122,123,124,125,126], sensors [65] and microfluidics [127,128,129]. Therefore, detailed descriptions of these technologies are not given in this review. In recent years, electrohydrodynamic (EHD) 3D printing based on near-field electrospinning (NFES) process has been increasingly applied in the fabrication of micro/nano objects using micro/nanofibers as building blocks [130]. This process has also been used in the fabrication of piezoelectric tactile sensors and is described in this review.

### 3.2. A Brief Overview of 3D Printing Applications in Tactile Sensors

To date, many researchers have use 3D printing as a powerful tool to address the challenges faced with tactile sensor fabrication. Usually, a tactile sensor is composed of several components including a sensing element, flexible substrate, flexible electrodes, an encapsulation and wiring. Not every tactile sensor has all of the components and the structures of tactile sensors vary from each other. In this review, the 3D printing applications in tactile sensors are categorized into five aspects according to the different types of 3D-printed components. 

(1) 3D-printed molds for microstructuring substrate, electrodes and sensing element. For this type of applications, 3D printing is not directly used to fabricate tactile sensor components but used to fabricate molds with various tailored surface structures. These surface structures are replicated by casting elastomeric materials to obtain microstructured substrates, electrodes or sensing elements. With these microstructured components, tactile sensor performance can be improved.

(2) 3D-printed flexible substrate or sensor body to accommodate sensing element. For this type of applications, 3D printing is used to directly fabricate flexible substrate or sensor body using elastomeric materials. Then, the printed substrate and sensor body is used to accommodate sensing element to form a tactile sensor. The major functional requirement for this type of applications is its flexibility and stretchability. To date, many soft silicone rubber-like 3D printable materials have been commercialized and applied to fabricate flexible substrate and sensor body. However, these commercial printable materials still have limited stretchability.

(3) 3D-printed sensing elements for tactile sensors. As for this type of applications, 3D printing is used to fabricate sensing elements with tailored geometries, shapes and microstructures to improve the performance of tactile sensors. Toward this goal, 3D printable sensing materials are required. Usually, these types of materials are not commercially available. Thus, many research groups have developed various types of printable sensing materials such as mixtures of silicone elastomer and conductive fillers as printable piezoresistive inks, printable silicone elastomers as dielectric layer for capacitive tactile sensor and printable piezoelectric polymers for piezoelectric tactile sensors.

(4) 3D-printed flexible electrodes for tactile sensors. In some cases, stretchable electrodes are essential to tactile sensors. For example, a capacitive tactile sensor is usually composed of a pair of conductive electrodes and a dielectric layer. Electrodes that are simultaneously electrically conductive and stretchable are highly desired. They should maintain low resistance change upon repeated stretching. 3D printing can be used to fabricate highly stretchable electrodes with a tuned structure such as serpentine design and excellent resistance stability upon stretching can be achieved.

(5) Fully 3D-printed tactile sensors. As for this type of applications, 3D printing is not used to print a single component, but used to print all the components incorporated in a tactile sensor by a specifically designed printing process. Some research groups have proposed such processes to fabricate a whole tactile sensor in one printing procedure.

Table 1 lists representative studies on 3D printing applications in tactile sensors. The 3D printing technology, sensing mechanism, measurable loads, printable materials, materials shape and fabricated components are given. Each study was categorized into these five types of applications according to the fabricated components via 3D printing. To date, the major 3D printing technologies that have been applied to the fabrication of tactile sensors include photopolymerization-based processes, fused deposition modeling (FDM), direct ink writing (DIW) and EHD 3D printing. The following sections describe the four types of 3D printing technologies and their applications in tactile sensors. Other 3D printing processes such as binder jetting, selective laser sintering/melting and direct energy deposition-based processes are seldomly used in tactile sensors and are not described in this review.

### 3.3. Photopolymerization-Based 3D Printing for Tactile Sensors

**Figure 3:**
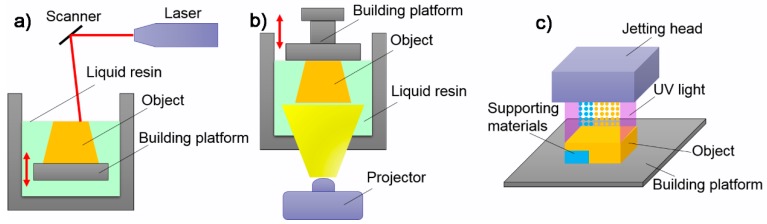
Photopolymerization-based 3D printing: (**a**) SLA; (**b**) DLP; and (**c**) PolyJet.

Photopolymerization-based 3D printing include four types of technologies: stereolithography (SLA), digital light projection (DLP), PolyJet and two-photon polymerization (2PP). SLA typically uses a scanning ultraviolet (UV) laser to cure photocurable resins to form 3D objects layer upon layer (Figure 3a). DLP uses a digital light processing unit (projector) that could enable the exposure and photocuring of a complete layer of resins to form 3D objects (Figure 3b). PolyJet was developed by Stratasys Objet Geometries Co, Ltd. (Eden Prairie, MN, USA) and it uses an inkjet printing-like jetting head with hundreds of micronozzles to eject photocurable resins and supporting materials. Each printed layer was cured by a UV light resource (Figure 3c). After printing, the supporting materials were removed to obtain the printed objects. As for 2PP, the photocuring process of materials was driven not by a single photon, but rather by two photons or multiple photons in combination. The process is typically used to fabricate complex 3D objects with micro/nano-sized features and seldomly used in 3D-printed tactile sensors.

**Figure 4:**
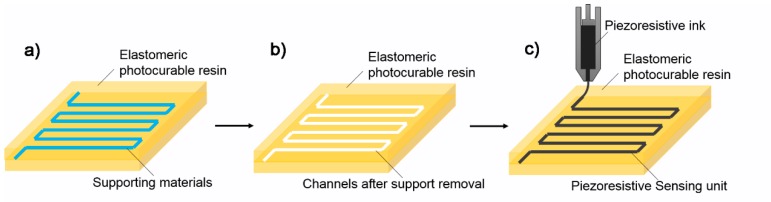
Indirect fabrication process for tactile sensors by photopolymerization-based 3D printing: (**a**) elastomeric sensor body with supporting materials; (**b**) elastomeric sensor body after support removal; and (**c**) piezoresistive ink injection or direct ink writing.

As for photopolymerization-based processes, the main machine and materials suppliers include 3D System, Formlabs, Stratasys Objet Geometries and Carbon 3D. Initially, these processes were only able to fabricate rigid and brittle parts and they are inappropriate for flexible devices. In recent years, several types of commercially available UV curable rubber-like or elastomeric materials were developed such as Carbon EPU40, Stratasys TangoPlus, Formlabs flexible, 3D System visiJet composite materials. These materials can be used to fabricate flexible and soft-touch prototypes. Some researchers attempted to use these commercially available 3D printing machines and flexible materials to fabricate tactile sensors. The fabrication process is shown in Figure 4. An elastomeric sensor body with supporting materials can be fabricated via SLA-, DLP- or PolyJet-based processes. Then the supporting materials were removed and the obtained channels were later filled with piezoresistive materials that functioned as the sensing element. After wiring, tactile sensors can be obtained. Alternatively, the piezoresistive ink can be patterned into the open microchannels using a direct ink writing process.

**Figure 5:**
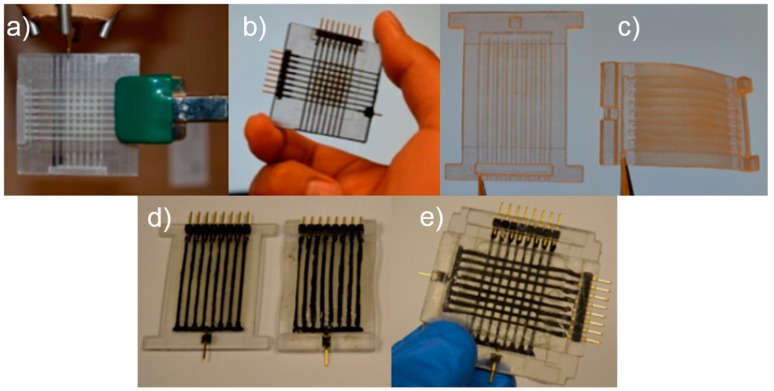
3D-printed sensor body using photopolymerization-based 3D printing: (**a**) elastomeric sensor body with internal channels; (**b**) tactile sensor with injection of piezoresistive ink; (**c**) top and bottom elastomeric sensor body; (**d**) direct ink writing of piezoresistive inks on sensor body; and (**e**) tactile sensor assembly [131]. Reproduced with permission. Copyright Spring Nature, 2015.

For example, Vatani et al. [131] used PolyJet-based process to fabricate 3D-printed tactile sensors. They designed a tactile sensor consisting of two sensing layers with straight piezoresistive line elements embedded in an elastomeric sensor body. The flexible sensor body was fabricated from a commercially available rubber-like photocurable resin TangoPlus using a PolyJet-based 3D printing machine (Connex 500, Stratasys Objet Co., Ltd., Rehovot, Isreal). Piezoresistive sensing materials were prepared by dispersing multi-walled carbon nanotubes (MWCNTs) into TangoPlus. Then, the obtained piezoresistive TangoPlus/MWCNTs composites were injected or directly printed into/onto the 3D-printed sensor body. The fabricated sensor body and tactile sensors are shown in Figure 5. Similarly, Agarwala et al. [132] used a commercial photopolymerization-based 3D printer (ProJet 5500X, 3D Systems Co., Ltd., Rock Hill, CA, USA) to fabricate a photopolymer matrix with embedded microchannels (500 μm diameter). In the printing process, the microchannels were initially filled with wax as the supporting materials and then washed away after printing. Then, the microchannels were injected with silver conductive ink to act as the piezoresistive sensing unit. The maximum tensile stress and strain were measured as 0.83 MPa and 18.2%, respectively. The gauge factor of the printed sensor was calculated to be 50 at compression strain of 1%.

**Figure 6:**
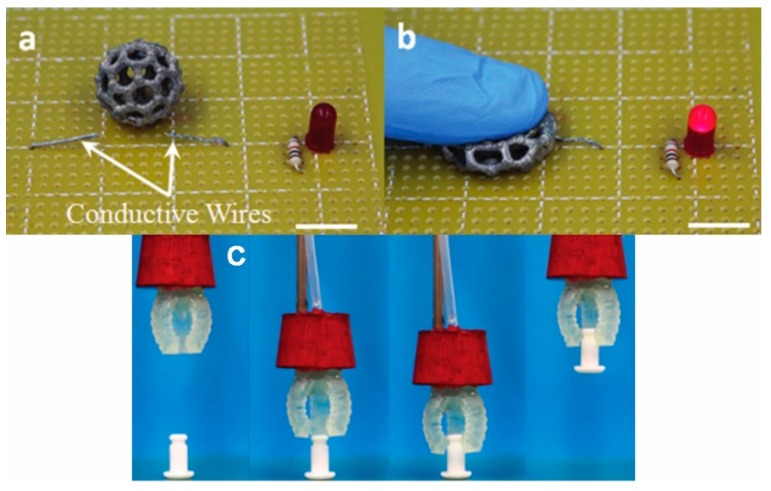
Highly stretchable elastomers fabricated by DLP: (**a**) DLP-printed Bucky ball coated with silver nanoparticles; (**b**) a LED turned on after pressing the Bucky ball; and (**c**) DLP-printed soft robotics [133]. Reproduced with permission. Copyright John Wiley and Sons, 2017.

The above studies demonstrated the applications of commercial UV curable elastomers in the fabrication of flexible tactile sensors. However, these elastomers still have great limitations such as insufficient stretchability and inappropriate mechanical properties. To address these issues, Patel et al. [133] developed a family of highly stretchable and UV curable elastomer systems that are suitable for photopolymerization-based 3D printing process including DLP, SLA and PolyJet. The elastomer was prepared by mixing a monofunctional monomer consisting of epoxy aliphatic acrylate (EAA), and a difunctional cross-linker consisting of aliphatic urethane diacrylate (AUD) diluted with 33 wt % of isobornyl acrylate (EAA-AUD mixture). The developed stretchable and UV curable elastomer was verified by DLP. The printed elastomer can be stretched by 1100% which was more than five times the elongation at break of commercial UV curable elastomers. In addition, the mechanical properties can be tuned for different applications by varying the EAA-AUD mixing ratio. The authors demonstrated the applications of the elastomer in DLP-printed Bucky ball electronic switches and soft actuators (Figure 6). In comparison with commercially available soft photopolymers, elastomeric sensor body with greatly improved stretchability can be directly fabricated using this new type of materials.

### 3.4. Materials Extrusion-Based 3D Printing for Tactile Sensors

**Figure 7:**
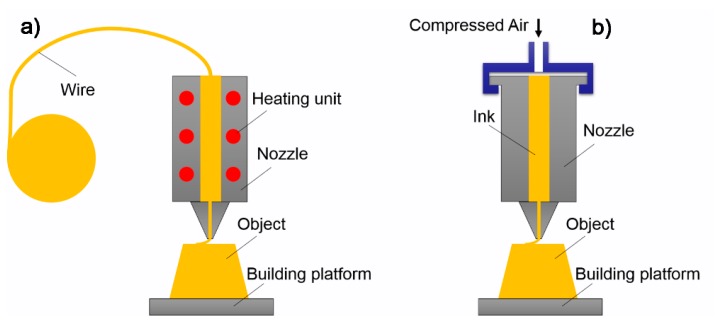
Materials extrusion-based 3D printing technologies: (**a**) FDM process; and (**b**) DIW process.

Fused deposition modeling (FDM) and direct ink writing (DIW) are the main two types of materials extrusion-based 3D printing processes (Figure 7). FDM typically uses a heated nozzle to melt polymer filaments to form 3D objects. While DIW uses a pneumatic nozzle or syringe nozzle to extrude printable inks with appropriate rheological properties to form 3D objects. Both processes have been used to fabricate tactile sensors. As for the FDM process, the printable materials are usually processed into filaments. This is a major limitation for its applications in the fabrication of tactile sensors. As for the DIW process, a wide range of printable inks can be tuned with appropriate rheological properties and offers a unique advantage for tactile sensor fabrication.

#### 3.4.1. FDM Process for Tactile Sensors

**Figure 8:**
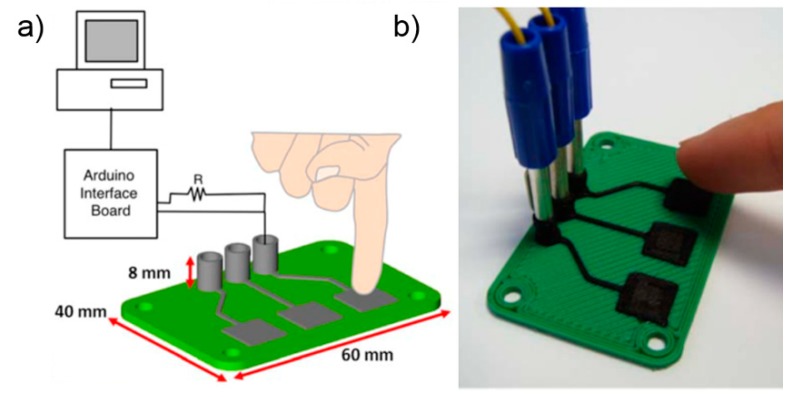
FDM-printed capacitive sensor-based HID using PCL/CB conductive composite: (**a**) schematic of the device; and (**b**) 3D-printed capacitive sensors [134].

The first FDM-printed tactile sensor was demonstrated by Leigh et al. [134] They fabricated flexible piezoresistive and capacitive sensors from polycaprolactone/conductive carbon black (PCL/CB) composite materials using a desktop FDM 3D printer. PCL/CB composites was processed into filaments with a diameter of 1.5 mm and extruded through a heated nozzle to obtain printed objects. The composite filaments showed piezoresistive properties due to the addition of conductive CB particles in the PCL matrix. A U-shape piezoresistive sensor was fabricated on a perspex substrate. With the flexing of the substrate, electrical resistance change of the printed sensor was observed. In addition, the authors fabricated a capacitive pressure sensor-based human interface device (HID) using FDM (Figure 8a,b). The HID device can be used to capture the touch sequence by detecting the pressure. These results showed that PCL/CB composites can be processed into filament-type materials for extrusion and tactile sensors may be fabricated via FDM process.

Saari et al. [135] used FDM process to fabricate capacitive force sensors consisting of 3D-printed transparent ABS-based upper and lower plates, two conductive electrodes and a dielectric layer. The dielectric layer was a 1.27 mm thick thermoplastic elastomer (TPE, Kraton G1643M, Kraton Performance Polymers Inc., Huston, TX, USA) capacitor core printed by FDM. The conductive electrodes were fabricated using a fiber encapsulation additive manufacturing (FEAM) process. In this process, bare copper wire with the diameter of 127 μm was encapsulated by TPE during the extrusion process. The sensitivity of the 3D-printed sensor was measured as 0.215 ± 0.002 pF/kN. The sensor showed good synchronization between the pressure loads and measured capacitance data. However, 8.3 s delay was observed due to materials hysteresis.

**Figure 9:**
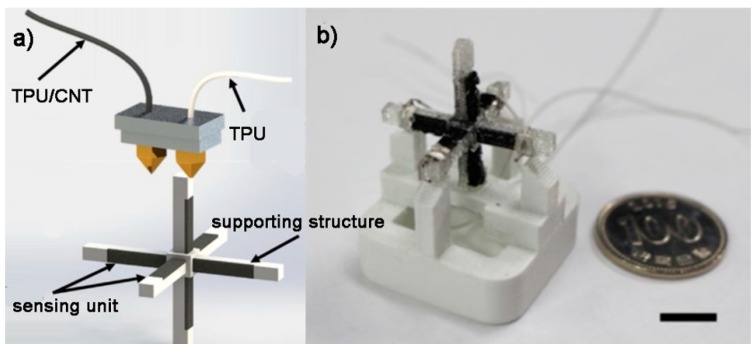
FDM 3D printing for multiaxial force sensors: (**a**) FDM process with dual nozzles to print TPU and TPU/CNT respectively; and (**b**) 3D-printed multiaxial force sensor [137]. Reproduced with permission. Copyright Elsevier, 2017.

Christ et al. [136] prepared thermoplastic polyurethane/multiwalled carbon nanotubes (TPU/MWCNTs) nanocomposite fibers that can be printed by FDM. A piezoresistive strain sensor with dimensions of 1.6 mm × 1.6 mm × 100 mm was fabricated. The failing strain was ~60% for the nanocomposites with MWCNTs content of 5% and a high gauge factor of 176 was obtained. These results demonstrated that FDM-printed TPU/MWCNTs strain sensor exhibited both excellent piezoresistive properties and good stretchability. Kim et al. [137] used a FDM 3D printer with a dual nozzle system that allow the printing of two different materials to fabricate 3D multiaxial force sensor (Figure 9). 3D cubic cross shaped sensors consisting of a structural support and a sensing part were fabricated. The structural part was printed with TPU filament and the sensing part was printed with TPU/CNT nanocomposite filament. The fabricated sensor was able to measure forces along three axes (*x*, *y* and *z*).

In addition to directly printing functional components for tactile sensors, FDM can also be used to fabricate molds for microstructuring sensing elements in an indirect approach. For example, Zhuo et al. [138] used a commercial FDM machine to print an ABS mold with tailored surface structures. Then, PDMS was cast onto the mold and microstructured PDMS film with periodical micro-grooves on the surface was obtained and used as the dielectric layer. A capacitive pressure sensor was fabricated by sandwiching the PDMS film by two ITO-coated PET films as electrodes. It was found that the assembled capacitive sensor achieved higher pressure sensitivity than using the micro-fabricated silicon wafer molds.

#### 3.4.2. Electric Poling-Assisted FDM Process for Tactile Sensors

**Figure 10:**
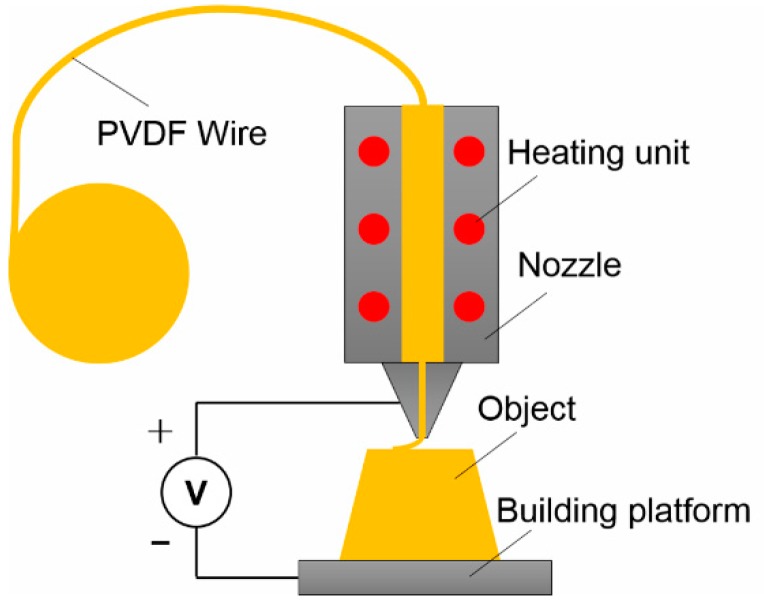
Electric poling-assisted FDM process for tactile sensors.

FDM can also be used to fabricate piezoelectric tactile sensors using electric poling-assisted FDM process (EPAM) (Figure 10). Lee et al. [139] proposed this new process by combining filament extrusion and applying high electric field between the nozzle tip and substrate. Using this process, piezoelectric polymers such as PVDF filaments can be directly printed into piezoelectric tactile sensors. When a high electrical field was applied to the molten polymer fiber between the nozzle tip and substrate, the PVDF polymer experienced a combinational process of drawing at high temperature, electric poling under high electrical field and annealing at high pressure that contributed to the formation of β phase crystalline structure during the printing process. The β phase is primarily responsible for the piezoelectric properties of PDVF polymer. The authors found that stronger electric field produced greater piezoelectricity in the printed structures. The measured output current was ±1.5 nA for a single printed layer.

However, the initial EPAM process was only capable of printing pure PVDF filaments and also limited to fabricate only a single layer. In addition, the applied electrical field intensity should not exceed 2 MV·m^−1^, otherwise it may cause the printer to lose communication with the computer. To overcome these limitations, Kim et al. [140] developed an enhanced EPAM process which could enable the application of higher electrical field up to 40 MV·m^−1^ and the fabrication of multiple layers. With improved electrical field intensity, the authors fabricated piezoelectric pressure sensors using composites of PVDF matrix and BaTiO_3_ (BTO) fillers to achieve better polarization and enhanced piezoelectric properties. The BTO/PVDF composites with different BTO loading content ranging from 3% to 15% were prepared. The authors measured the piezoelectric coefficients of nonpoled PVDF, poled PVDF and BTO/PVDF composite with different BTO content. It was found that the highest amount of 55.91% β phase PVDF was obtained when the BTO content was 15%. The piezoelectric coefficient of 15 wt % BTO/PVDF composite was 0.101 pC/N and ~1300% larger than nonpoled PVDF. Results showed that BTO filling and in-situ poling could effectively improve the piezoelectric properties. 

#### 3.4.3. Direct Ink Writing for Tactile Sensors

As for the DIW process, the preparation of a printable ink is a crucial step. A printable ink is usually endowed with appropriate rheological properties and extruded through a nozzle tip using pressurized air or a syringe. The shape and mechanical integrity of printed structures must be maintained during the printing process. Toward this goal, many researchers have developed various printable inks. Most of the printable inks for tactile sensors are mixtures of conductive fillers and stretchable elastomers. Conductive fillers include carbon-based nanomaterials (carbon black, carbon nanotubes, graphene, etc.) and metal-based nanomaterials (metal nanoparticles and nanowires). Silicone elastomers such as Ecoflex and PDMS are the most commonly used matrix materials. In addition, the concept of “ionic skin” using stimuli-responsive hydrogels to fabricate flexible tactile sensors with unique biocompatibility was proposed. DIW can also be used to fabricate ionic tactile sensors using biocompatible hydrogels as printable inks.

In conventional DIW process, printable inks are directly deposited onto the substrate. Some research groups proposed different forms of DIW processes. For example, printable inks can be deposited into a reservoir filled with a second medium. For tactile sensors, piezoresistive inks can be directly printed into a silicone elastomer reservoir and encapsulated within the elastomeric matrix immediately. This process was named embedded DIW process. Another different form of DIW process is the multicore-shell DIW process. In this process, multiple layers of printable inks including a core, middle layers and an encapsulation layer can be coextruded through a nozzle to form a multi-layered tactile sensor in one printing procedure. All the processes have been applied to the fabrication of tactile sensors.

(1) Conventional DIW Process

**Figure 11:**
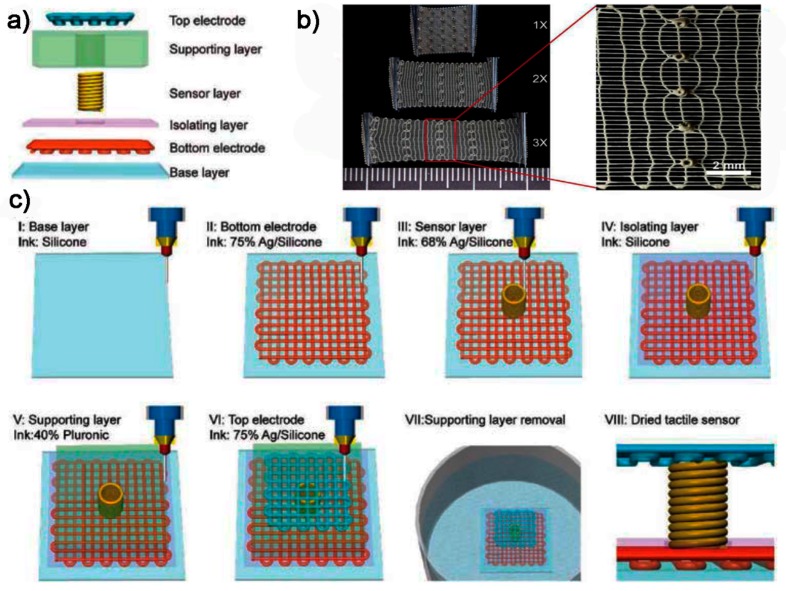
Tactile sensor design and 3D printing process: (**a**) schematic of the tactile sensor design; (**b**) stretchable tactile sensor fabricated via DIW; and (**c**) 3D printing procedures [141]. Reproduced with permission. Copyright John Wiley and Sons, 2017.

Guo et al. [141] proposed a piezoresistive tactile sensor design composed of the base layer, bottom electrode, isolating layer, supporting layer, sensing layer and top electrode. All of the components in this tactile sensor are fabricated via DIW. The base layer and isolating layer were fabricated using modified silicone ink: DragonSkin 10 with the addition of a thickening agent and curing retarder. The top and bottom electrodes were fabricated from mixtures of silicone elastomer and silver nanoparticles (AgNP) as printable inks. The electrical conductivity of the silicone/AgNP ink increased with the loading of AgNP and the percolation threshold was found to be 67.45%. The top and bottom electrode was printed with 75 wt % AgNP/silicone inks given the good balance of high conductivity and stretchability. The sensing layer was printed with 68 wt % AgNP/silicone ink since it has the best piezoresistive properties. The supporting layer was printed using a 40 wt % pluronic ink and later washed away by water. The fabrication process is shown in Figure 11. As for the sensing layer, the current change (*ΔI*/*I*_0_) was measured to be 2500%, 8000% and 17,000% under 60, 120 and 250 KPa cyclic pressure loads, respectively. For the top and bottom electrodes, the current change was 280% when 250 KPa pressure load was applied. The printed sensors showed good linear current–voltage characteristics. The gauge factor of the entire printed device was found to be ~180. As for the frequency response, hysteresis was negligible when the frequency was below 0.25 Hz, while a relative hysteresis of 82% was observed when the frequency was 1 Hz due to the viscoelastic properties of silicone. The authors also demonstrated the applications of printed sensors in measuring heartbeat, finger pressure on keyboard and pressure distribution based on sensor arrays. 

Besides the commonly used materials prepared from the mixture of conductive fillers and elastomeric matrix, Kim et al. [142] developed a new carbon-based 3D printable composite dough materials for flexible sensors. The composite dough materials are comprised of an electrostatically assembled carbon composite and a thermoplastic triblock-copolymer elastomer, polystyrene-polyisoprene-polystyrene (SIS). To obtain a tailored spatial distribution of the conductive network, the authors modified multi-walled carbon nanotubes (MWCNTs) chemically to have surface-amine functional groups (NH_2_-MWCNTs). By introducing the surface-amine functional groups, nanotubes were confined to the surface of graphene oxides (GOs), thereby generating an electrostatically assembled carbon composite. Then the 3D printable dough materials were prepared by mixing the NH_2_-MWCNTs/GO composite fillers with SIS elastomer matrix. The authors firstly measured the piezoresistive response of GO-free MWCNTs/SIS composites. It was found that the use of MWCNTs alone was not appropriate for fabricating highly sensitive strain sensors, although excellent stretchability was achieved. By introducing GOs, only a slight amount of GOs incorporation (NH_2_–MWCNTs/GO ratio = 450) resulted in a dramatic increase of gauge factor, however the linearity and hysteresis behavior were inferior. When NH_2_-MWCNTs/GO ratio was 9, a gauge factor of 72 was obtained. Meanwhile, excellent linearity of 0.94 and reduced hysteresis were achieved. By tuning the compositions of dough materials developed in this study, the sensor performance can be easily adjusted. It was also shown that the fabricated sensor was able to detect strains as low as 1%.

**Figure 12:**
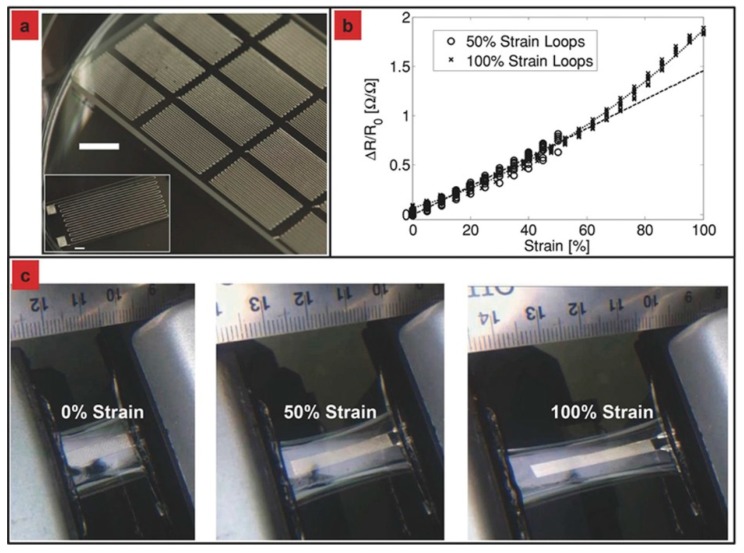
3D-printed Ga-In alloy/PDMS strain sensor using DIW: (**a**) 3D-printed Ga-In alloy structures on glass; (**b**) resistance change with strain; and (**c**) strain sensor encapsulated in PDMS. Reproduced with permission. Copyright John Wiley and Sons, 2014.

DIW process also can be used to print liquid metals. For example, Boley et al. [143] developed a direct ink writing method to print gallium-indium (Ga-In) alloy on two types of substrates: glass and PDMS. It was found that the printed structures were able to maintain their shape due to the formation of gallium-oxide skin during the printing process. Strong adhesion between Ga-In alloy and substrate also facilitated the direct writing of Ga-In alloy. The authors directly printed Ga-In alloy onto PDMS substrate to obtain a strain sensor (Figure 12). The sensor showed good linearity between resistance change and strain (below 50%) and a gauge factor of 1.5 was achieved.

**Figure 13:**
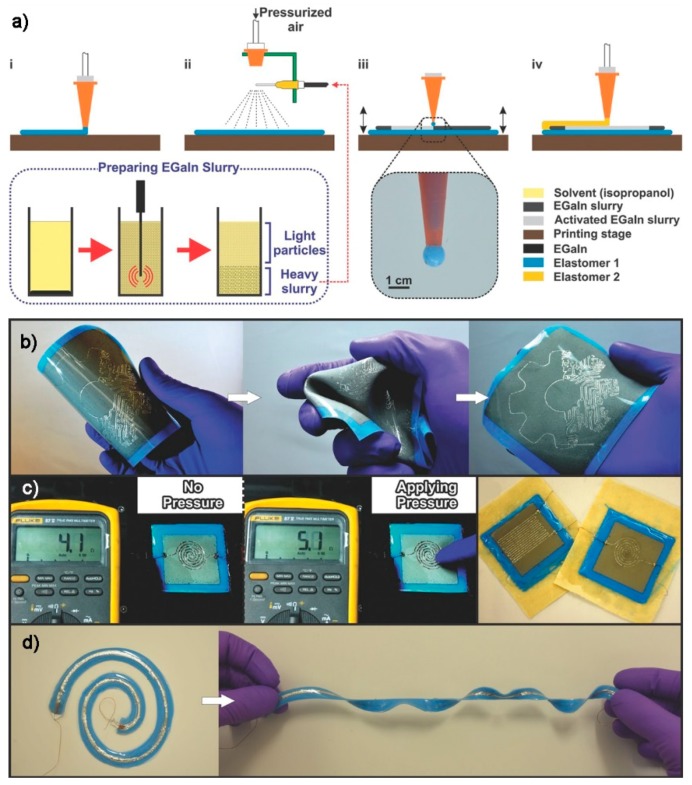
All-printed stretchable pressure and strain sensors based on liquid metals [144]: (**a**) the fabrication process; (**b**) printed complex electronics with excellent flexibility; (**c**) printed pressure and strain sensors; and (**d**) printed stretchable coil sensor. Reproduced with permission. Copyright John Wiley and Sons, 2017.

Further, the research group developed a sequence of procedures toward all-printed flexible and stretchable tactile sensors [144]. The fabrication process (Figure 13a) included four steps: (i) printing of a base elastomer on the stage using DIW process; (ii) spray printing of liquid metal slurry (Ga-In alloy) using pressurized air; (iii) selective activation of the electrical path; and (iv) encapsulation by an elastomer to seal the device using DIW. During the spray printing of liquid metals, oxide shells formed around the deposited liquid particles. Therefore, the authors developed a selective activation process to rupture the oxide shells and merge the liquid particle cores to create an electrically conductive path by a pressure between the tapping stage and nozzle tip. It was demonstrated that printed electronics with complex geometries can be fabricated via this process. For example, the authors fabricated coil-shaped pressure sensor and serpentine-structured strain sensor with excellent stretchability and flexibility (as shown in Figure 13b–d). This new technology provides a feasible method for the fabrication of all-printed tactile sensors based on liquid metals.

**Figure 14:**
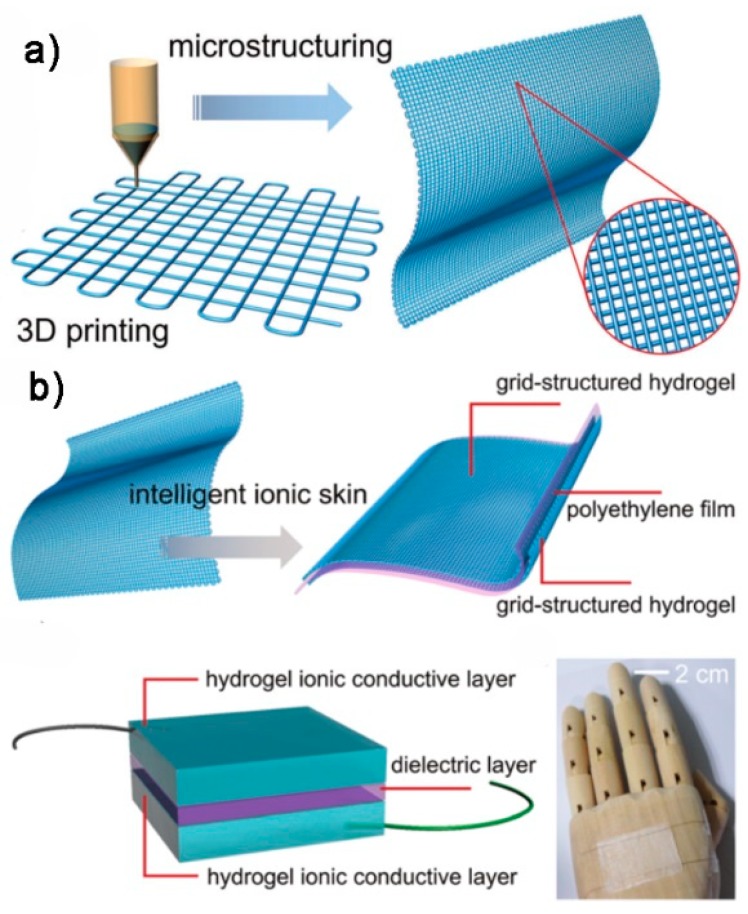
3D-printed ionic tactile sensors: (**a**) 3D-printed grid-structured hydrogel film; and (**b**) tactile sensor structure and assembly [145]. Reproduced with permission. Copyright Royal Society of Chemistry, 2017.

Conventional flexible tactile sensors are usually fabricated from organic elastomer matrices embedded with inorganic conductive materials and they usually detect signals through electronic conductors. In recent years, the concept of “ionic skin” using soft, biocompatible and ionically conductive hydrogels was proposed. The unique advantage of “ionic skin” is its biocompatibility with human tissue. Inspired by the idea of “ionic skin”, Lei et al. [145] developed a capacitive multifunctional and flexible skin-like sensor by incorporating 3D printed thermo-responsive hydrogel into a capacitor circuit. The printed capacitive sensor was comprised of three layers, a polyethylene film as the dielectric layer sandwiched by two 3D-printed grid-structured thermo-responsive hydrogels as ionically conductive layer (Figure 14a,b). The capacitive sensor was able to output stable and sensitive capacitance-temperature response and also showed very high pressure sensitivity within 1 KPa. A pressure sensitivity of 0.45 KPa^−1^ was achieved using the grid-structured hydrogel. The authors demonstrated its applications in detecting body temperature, gentle finger touches and finger bending motions. The combination of stimuli-responsive hydrogels and 3D printing may eventually lead to various multifunctional ionic sensors for wearable electronics.

Another example using stimuli-responsive hydrogels for 3D-printed flexible sensors was demonstrated by Liu et al. [146] They prepared a double-network hydrogel by combining an ionically cross-linked κ-carrageenan network with a covalently cross-linked polyacrylamide (PAAm) network. It was found that the warm pregel solution of *k*-carrageenan/AAm had the required rheological properties for DIW. The printed structures have remarkable mechanical strength and self-healing properties after UV exposure. In addition, the hydrogel was also found to have good piezoresistive properties that is essential to strain sensors. The gauge factor of the double-network hydrogel was measured to be 0.23 at the strain of 100% and 0.63 at the strain of 1000%. The authors also demonstrated the double-network hydrogel-based strain sensors in monitoring human finger bending motion.

**Figure 15:**
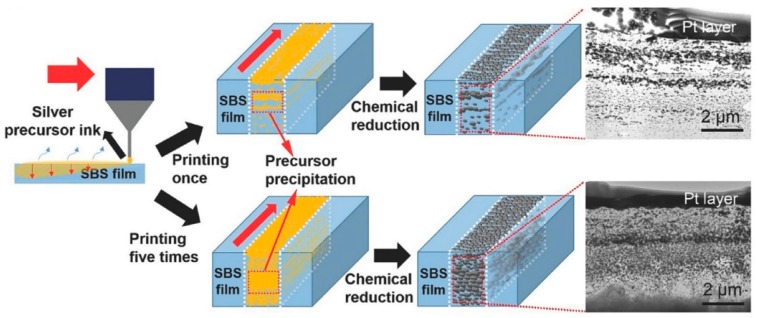
Printing metal precursor solution onto stretchable substrate to fabricate surface embedded conductive electrodes [147]. Reproduced with permission. Copyright John Wiley and Sons, 2017.

In addition, DIW also can be used to print metal precursor solution on a stretchable substrate. After chemical reduction of precursor into metal nanoparticles, stretchable conductive electrodes with surface embedded metal particles can be obtained (Figure 15). Song et al. [147] developed a silver precursor (silver trifluoroacetate) solution and then the precursor solution was printed onto a poly(styrene-*b*-butadiene-*b*-styrene) (SBS) film substrate. A penetration process of precursor solution into the SBS substrate took place only a few seconds after printing. Then, a chemical reduction process by hydrazine vapor was applied to convert silver precursor into silver nanoparticles. It was found that highly conductive patterns with the thickness of 60 μm could be easily obtained after repeated printing. The surface embedded conductive silver nanoparticles in the substrate can offer excellent stretchability and strong interfacial connections between conductive patterns and substrate. In comparison with conventional printing process, the interfacial connections between conductive materials and substrates are usually weak. The authors measured the resistance change of printed conductive line patterns at various strains. It was shown that the printed electrodes can not only be used as piezoresistive sensing element, but also stretchable, conformal and conductive electrodes.

Silver nanoinks have also been used as printable inks for piezoresistive strain sensors. For example, Zhang et al. [148] printed silver nanoparticle-based inks on a polyurethane acrylate (PUA) substrate. Serpentine structured patterns were printed with different curvature radiuses. It was found that the stretchability and resistance change were strongly dependent on curvature radius. Serpentine structures with curvature radius of 200 μm exhibited a record high gauge factor of ~10^7^ at strain of ~12%. The increase of curvature radius resulted in higher stretchability and reduced gauge factor. The authors also demonstrated the printed strain sensors in detecting finger motion. Cai et al. [149] developed a printable silver nanowire-based ink that can be printed on various substrates including silicon wafer, PDMS and glass. The authors studied the variation of resistance change with different average nanowire lengths of 4.4, 15.6, 38.5 μm. It was found that longer nanowire length had better capability to maintain effective conductive network upon stretching. Meanwhile, smaller resistance change with applied strain was observed (1000% resistance change for 4.4 μm nanowire length and 90% for 38.5 μm at the strain of 20%). The silver nanowires on PDMS substrate can offer both excellent stretchability and conductive properties, therefore can be used as stretchable electrodes. The authors demonstrated a sandwich-structured capacitive pressure sensor composed of two printed stretchable electrodes and a silicone elastomer dielectric layer. The pressure sensor showed a pressure sensitivity of 0.106 KPa^−1^ below 1 KPa and a minimum detectable pressure of ~100 Pa.

**Figure 16:**
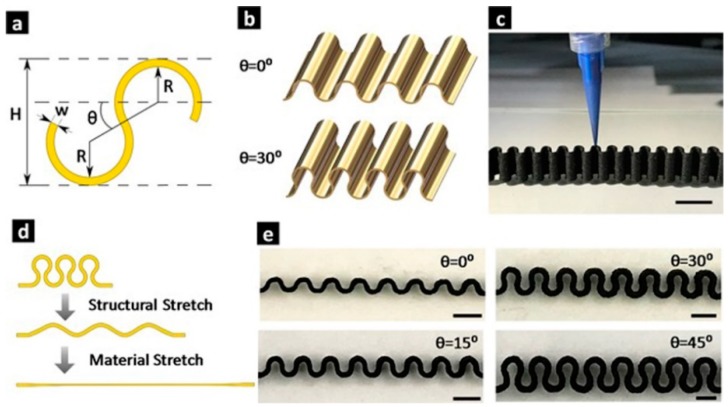
3D-printed wavy-structured stretchable electrodes with different structural parameters. (**a**) Cross-sectional view of the serpentine design; (**b**) 3D schematics of the serpentine electrodes with θ = 0°/30° (fixed *R*, *w*); (**c**) photograph showing the 3D printing process. Scare bar 10 mm; (**d**) schematics showing the unique two-degree stretching process of the serpentine design; (**e**) cross-sectional photographs of the 3D printed wavy electrodes with different joining angles. Scare bar 5 mm. Reproduced from [150] with permission. Copyright John Wiley and Sons, 2017.

In previously mentioned studies, various type of printable inks were developed to fabricate tactile sensors via DIW process including silicone/Ag composites, NH_2_-MWCNTs/GO/SIS composites, liquid metal-based inks, biocompatible hydrogels, metal precursor and silver nanoinks. It can be seen that DIW is a facile, simple and versatile technology with the fewest restrictions on printable materials. Meanwhile, DIW can also be used to fabricate tactile sensor components into tailored structures with tunable stretchability and electrical conductivity. For example, Wei et al. [150] printed free-standing wavy-structured stretchable electrodes based on a PDMS/MWCNTs composite ink. These electrodes were designed and fabricated with different structural parameters (Figure 16). The stretchability and resistance change upon repeated stretching were measured. It was found that different stretchability and resistance change properties were obtained for electrodes with different structural parameters. Through structural optimization, both high stretchability (300%) and high resistance stability (5% resistance change at 100% strain) can be achieved.

(2) Embedded DIW for Tactile Sensors

**Figure 17:**
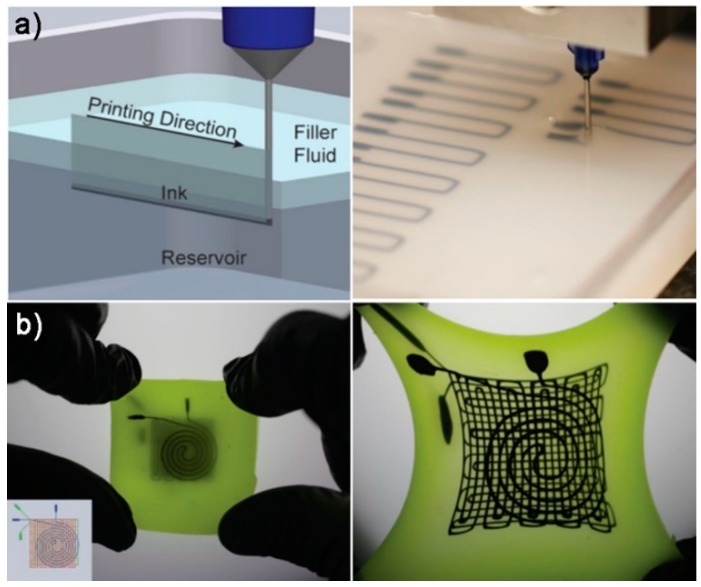
Embedded DIW process for tactile sensors: (**a**) embedded direct ink writing; and (**b**) fabrication of a flexible strain sensor in unstrained state and stretched state [151]. Reproduced with permission. Copyright John Wiley and Sons, 2017.

In the above DIW processes, the prepared inks were directly printed onto a flexible or stretchable substrate mounted on the building platform. Alternatively, inks can also be printed into a fluid medium reservoir and directly encapsulated in the fluid medium. Muth et al. [151] developed embedded DIW process to fabricate piezoresistive flexible strain sensors. In this process, conductive piezoresistive inks were directly printed within an elastomeric reservoir and encapsulated by a filler fluid (Figure 17a). Conductive carbon grease—a suspension of carbon black particles in silicone oil—was used as printable inks for the sensing element. Modified commercial silicone elastomer (uncured Ecoflex 00-30, Smooth-on Inc., Macungie, PA, USA) was used as the reservoir and filler fluid. After printing, the reservoir and filler fluid were *co*-cured to form a monolithic part with piezoresistive conductive ink encapsulated in the elastomeric matrix. Owing to the excellent stretchability of elastomeric matrix and conductive ink, the 3D-printed sensor could endure strains up to 700~800% mechanically and exhibit predictable electrical responses up to ~400% strain (Figure 17b). The gauge factor of these flexible strain sensor was 3.8 ± 0.6 that was similar as conventional metallic strain gauges. The authors fabricated a glove with 3D-printed strain sensors encapsulated in elastomer for each finger that could be used to monitor the digit motion in real time. However, significant hysteresis was observed during cycling due to the inherent hysteresis associated with the elastomeric loading.

(3) Multicore-Shell DIW for Tactile Sensors

**Figure 18:**
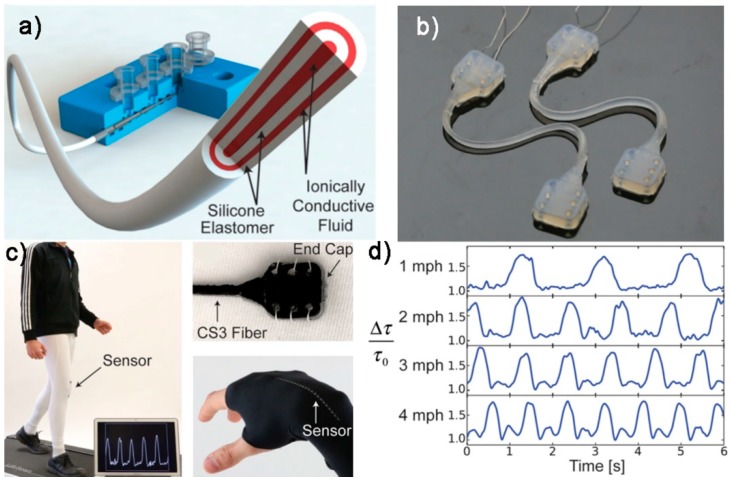
(**a**) Coextrusion of multiple inks to fabricate fiber-shaped multi-layered capacitive sensors; (**b**) the assembled sensor; (**c**) textile-mounted sensor for walking detection; and (**d**) the decay time response [152]. Reproduced with permission. Copyright John Wiley and Sons, 2017.

Another unique advantage of DIW process is its ability to enable the coextrusion of multiple inks to form multiple-layered fiber-shaped structures. For example, Frutiger et al. [152] developed a multicore-shell printing process to fabricate textile-mounted, capacitive flexible strain sensors. Ionically conductive ink composed of glycerol, sodium chloride, and polyethylene glycol was prepared and acted as the conductive layer. A commercially available silicone elastomer Dragonskin 10 was used as the dielectric layer. The fiber-shaped sensor consisted of four concentric layers including a core conductive fluid layer, dielectric silicone elastomer layer, an outer conductive fluid layer and an encapsulation silicone elastomer layer (Figure 18a,b). The four layers were coextruded through a customized printing head and deposited to form a capacitive fiber sensor. The authors measured the capacitance, resistance and decay time responses as a function of strain. It was demonstrated that the sensor could output any of these three quantities: capacitance, resistance and decay time. The gauge factor for the capacitive response of the sensor was 0.348 ± 0.11. The dynamic capacitance response was also measured for different strain amplitudes and frequencies. It was shown that the sensor output could accurately track the strain input. The authors also mounted the multicore–shell fiber sensor onto textiles across the knee by sewing and measured the decay time output for different walking speeds from 1 to 4 mph (Figure 18c,d). It was demonstrated that the fiber sensor could be used to detect the walking gait.

### 3.5. Electrohydrodynamic 3D Printing for Piezoelectric Tactile Sensors

**Figure 19:**
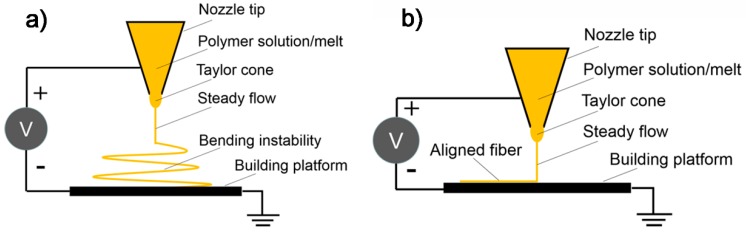
(**a**) Far-field electrospinning; and (**b**) near-field electrospinning.

EHD 3D printing is an emerging micro/nano fabrication technology based on electrostatic force-induced jetting flow of materials to fabricate micro/nanofibers. EHD 3D printing derives from a classical electrohydrodynamic flow jetting process: electrospinning which was developed as an important fiber production technology (Figure 19a). In this process, the polymer solution/melt jets generated from the Taylor cone undergo two stages. In the first stage, the polymer jets travel in a straight line and then goes through a “whipping process”. Finally, the polymer jets elongate and deposit onto the building platform to form micro/nanofibers. Conventionally, the distance from nozzle tip to building platform was 5–20 cm and the inherent instability of electrospinning made the process controllability very poor. Using conventional electrospinning, nanofiber arrays of piezoelectric polymers such as PVDF and PVDF-TrFE can be fabricated and used as the piezoelectric sensing unit for tactile sensors. The applied high electrical fields for poling can greatly enhance the piezoelectric properties. However, the fiber alignment and orientation are difficult to control. Sun et al. proposed a near-field electrospinning (NFES) process that utilized the steady polymer jet region to achieve controlled deposition by reducing the distance between nozzle tip and building platform to 0.5–3 mm (Figure 19b). The modified process can be used to produce user specific patterns of micro/nanofibers in a simple and highly-efficient manner. By integrating the controlled deposition of NFES process and the layer-by-layer manufacturing principle of 3D printing, micro/nano 3D printing can be achieved.

**Figure 20:**
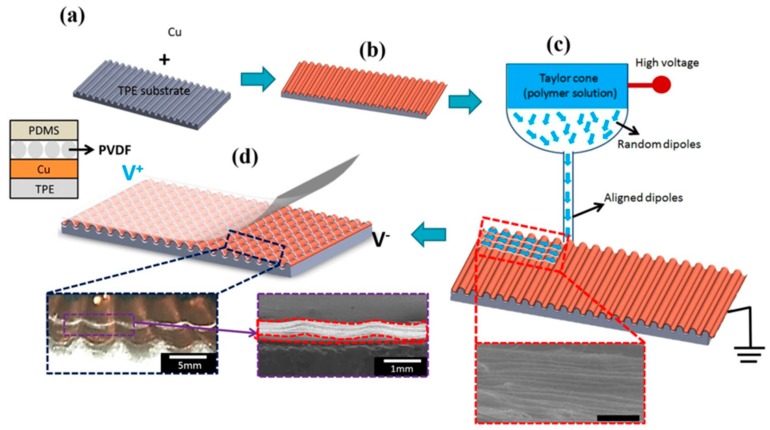
EHD printing of aligned PVDF micro/nanofibers on FDM-fabricated wavy structures: (**a**) FDM-fabricated TPE wavy substrate; (**b**) Cu foil adhesion on wavy substrate; (**c**) NFES of PVDF on wavy substrate; and (**d**) encapsulation with PDMS [153].

Using EHD 3D printing, Fuh et al. [153] fabricated piezoelectric pressure sensors by depositing piezoelectric PVDF onto FDM-fabricated wavy substrates (Figure 20). Firstly, a thermoplastic elastomer substrate with topologically tailored wavy structures was fabricated via FDM process. Secondly, a copper foil was attached to the wavy substrate. Then, polyvinylidene fluoride (PVDF) micro/nano fibers were deposited onto the substrate surface by near-field electrospinning (NFES). Finally, the sensor was encapsulated by a PDMS film to form a piezoelectric pressure sensor. By using NFES, highly-aligned piezoelectric fiber array with diameter of 1–5 μm was obtained. The authors fabricated three types of substrate including planar surface, square surface and sinusoidal surface structured surfaces. A total of 600 PVDF fibers were deposited and the output voltage was 2, 3.5 and 3.5 V, respectively, for three types of wavy structured surfaces. The results showed that the sinusoidal surface had the best output, possibly due to the largest total length of PVDF fibers. The authors also demonstrated the application of the fabricated sensor in foot pressure measurement and human motion monitoring.

In addition to piezoelectric polymer, Qin et al. [154] used EHD 3D printing to fabricate coplanar capacitive touch sensors based on a composite ink composed of 20–30 wt % Ag nanoparticles and triethylene glycol. The capacitive sensor was composed of two coplanar interdigitate microelectrode arrays printed on flexible PET film. The capacitance of the printed sensor was proportional to the number of electrodes. The authors measured the effects of finger touch on capacitance and it was demonstrated that the proposed sensor can be applied in touch displays.

### 3.6. Comparison of Different 3D Printing Technologies for Tactile Sensors

The previous section describes the 3D printing applications in tactile sensors. As for photopolymerization-based processes, the printable materials must be photocurable. Therefore, these processes are mainly limited to the fabrication of stretchable substrate and sensor body. Commercially available silicone rubber-like photopolymers can be directly used to fabricate flexible substrate and sensor body; however, they have limited stretchability. Elongation at break of ~1100% that was comparable to the most stretchable silicone elastomer was achieved for recently developed UV curable elastomers. These progresses will finally lead to the development of 3D printable highly stretchable photopolymers with sufficient stretchability for tactile sensors. In addition, a unique advantage is the high precision and complexity that can be achieved through photopolymerization-based processes. However, available stretchable photopolymers are still very limited. With novel photocurable elastomeric materials being developed, photopolymerization-based processes may have wider applications in tactile sensors. For example, mixtures of stretchable photopolymers and conductive materials may be applied to the fabrication of stretchable electrodes and sensing element.

As for the FDM process, a unique advantage is its simplicity and low-cost. However, the printable materials must be processed into filaments. This has been a limitation for its applications in tactile sensors. To date, only a few types of materials are available for FDM. Thermoplastic elastomer such as TPU was used to fabricate wavy substrate and dielectric layer for tactile sensors. Composite materials of thermoplastic polymer and conductive filler such as PCL/CB and TPU/MWCNTs were also fabricated into piezoresistive sensing element by FDM. In addition, electric poling-assisted FDM process was developed to print piezoelectric polymers such PVDF into piezoelectric sensing element. However, the major limitation of this process is the limited stretchability of printable materials (failing strain of 60% for 5 wt % MWCNTs/TPU composites).

As for direct ink writing, by dispersing conductive fillers such as carbon black, MWCNTs, graphene, metal nanoparticles or nanowires into elastomeric matrices such as silicone elastomer, various type of printable composite inks can be obtained. By tuning the rheological properties, these printable inks can be used to fabricate stretchable substrate, electrodes and sensing element. Using multicore-shell printing and multi-material printing, fully 3D-printed tactile sensors can be achieved in a single printing procedure. An unrivaled advantage of DIW process is its wide range of printable materials and its capability to extrude silicone elastomer with the largest stretchability. In addition, printable ionic conductive hydrogels have also been demonstrated to fabricate ionic tactile sensors by DIW process. All these advantages will make DIW a competitive 3D printing technology for tactile sensor fabrication.

As for EHD 3D printing, NFES-based process has the capability of depositing piezoelectric polymer solution into micro/nanofibers with improved piezoelectric properties. This process provides a new way to fabricate highly-oriented micro/nanofiber arrays with excellent fiber alignment and may find important applications in piezoelectric tactile sensors.

## 4. Conclusions and Outlook

This review summarizes the latest advances in the fabrication of tactile sensors by 3D printing. In comparison with other fabrication methods such as lithography-based process, physical/chemical vapor deposition and various chemical processes, 3D printing can construct complex 3D geometries with customized topological features in a highly efficient and low-cost manner. Topologically optimized tactile components including substrate, electrodes and sensing element with tailored microstructures can be easily fabricated. Higher sensitivity, fast response, smaller minimal detectable loads and better flexibility can be achieved through structural optimization. This advantage will make 3D printing play a more important role in advancing wearable electronics and electronic skin. As for different types of 3D printing technologies, there are different requirements on printable materials. Photocurable polymers are required for photopolymerization-based 3D printing. Filament type materials are required for FDM process. The rheological properties of printable inks should be tuned for DIW process. These restrictions have limited the amounts of available printable materials. In the future, novel printable materials with improved stretchability need further development. In addition, some research groups have proposed multi-material 3D printing processes that can deposit different materials in one printing procedure to achieve fully 3D-printed tactile sensors. It can be expected that more multi-material printing technologies will emerge and these processes will greatly accelerate 3D printing applications in tactile sensors. Moreover, multifunctional tactile sensors that can detect not only force-related information but also temperature, humidity, etc. have been under development. In the future, it can be anticipated that 3D-printed multifunctional tactile sensor may become a research focus. Finally, inspired by the concept of ionic skin, 3D-printed ionic tactile sensors using biocompatible hydrogels also have good prospects in the future.

## Figures and Tables

**Table 1:** 3D printing applications in tactile sensors.

3DP Technology	Sensing Mechanism	Loads	Printable Materials	Materials Shape	Printed Component	Ref.
PolyJet	Piezoresistive	Pressure	Photocurable TangoPlus	Photopolymer, photopolymer composites	Sensor body	[131]
	Piezoresistive	Pressure	Conductive photocurable TangoPlus/MWCNTs composites		Sensing element	[131]
	Piezoresistive	Pressure	Photocurable VisiJet composites		Sensor body	[132]
DLP	——	——	UV curable EAA/AUD stretchable elastomer		Substrate/Sensor body	[133]
FDM	Piezoresistive	Pressure	Conductive PCL/CB composite	Filament	Sensing element	[134]
	Piezoelectric	Pressure	Thermoplastic elastomer		Sensor wavy substrate	[153]
	Piezoresistive	Tensile strain	Conductive TPU/MWCNTs nanocomposites		Sensing element	
	Capacitive	Pressure	Thermoplastic elastomer		Dielectric layer	[135]
	Piezoresistive	Pressure	Supporting structure: TPUSensing element: TPU/MWCNTs composites		Supporting structure, sensing element	[137]
	Capacitive	Pressure	ABS		Molds for microstructuring sensing element	[138]
Electric poling-assisted FDM	Piezoelectric	Tensile strain	Piezoelectric PVDF polymer	Filament	Sensing element	[139]
	Piezoelectric	Pressure	Piezoelectric PVDF polymer	Filament	Sensing element	[140]
	Piezoelectric	Pressure	PVDF/BaTiO_3_ composites	Filament	Sensing element	[140]
Direct ink writing	Piezoresistive	Pressure	Sensing layer: 68 wt % Ag/silicone ink Electrode layer: 75 wt % Ag/silicone ink Substrate layer: silicone elastomer Supporting layer: 40% pluronic ink	Inks	Fully 3D-printed tactile sensor	[141]
	Piezoresistive	Tensile strain	Composite dough materials: NH_2_-MWCNTs/GO/SIS composites	Ink	Sensing element	[142]
	Piezoresistive	Tensile strain	Gallium-indium alloy	Ink	Sensing element	[143]
	Piezoresistive	Pressure, tensile strain	Elastomer, gallium-indium alloy	Ink	Fully 3D-printed tactile sensor	[144]
	Capacitive	Pressure, temperature	Thermo-responsive hydrogel	Inks	Ionically conductive layer	[145]
	Piezoresistive	Tensile strain	κ-carrageenan/PAAm double-network hydrogel	Inks	Sensing element	[146]
	Piezoresistive	Tensile strain	Silver precursor (silver trifluoroacetate) solution in alcohol or acetone	Inks	Stretchable electrodes, sensing element	[147]
	——	Tensile strain	PDMS/MWCNTs composite	Inks	Stretchable electrodes	[150]
	Piezoresistive	Tensile strain	Silver nanoparticle-based inks	Inks	Sensing element	[148]
	Piezoresistive	Tensile strain, pressure	Silver nanowire-based inks	Inks	Sensing element, stretchable electrodes	[149]
Embedded direct ink writing	Piezoresistive	Tensile strain	Suspensions of CB in silicone oil	Inks	Sensing element	[151]
Multicore-shell direct ink writing	Capacitive	Tensile strain	Conductive layer: ionically conductive ink composed of glycerol, NaCL, and PEG Dielectric/encapsulation layer: modified silicone elastomer	Inks	Fully 3D-printed tactile sensor	[152]
electrohydrodynamic printing	Piezoelectric	Pressure	Piezoelectric PVDF polymer	Polymer solution	Sensing element	[153]
	Capacitive	Finger touch	20 wt %–35 wt % Ag nanoparticles/triethylene glycol composite	Composite solution	Conductive electrode	[154]
